# Detecting KPC-2 and NDM-1 Coexpression in Klebsiella pneumoniae Complex from Human and Animal Hosts in South America

**DOI:** 10.1128/spectrum.01159-22

**Published:** 2022-08-18

**Authors:** Felipe Vásquez-Ponce, Karine Dantas, Johana Becerra, Gregory Melocco, Fernanda Esposito, Brenda Cardoso, Larissa Rodrigues, Keila Lima, Aline V. de Lima, Fábio P. Sellera, Renata Mattos, Lucas Trevisoli, Marco A. Vianello, Thais Sincero, Jose Di Conza, Eliana Vespero, Gabriel Gutkind, Jorge Sampaio, Nilton Lincopan

**Affiliations:** a Department of Microbiology, Institute of Biomedical Sciences, Universidade de São Paulo, São Paulo, Brazil; b Department of Clinical Analysis, School of Pharmacy, Universidade de São Paulo, São Paulo, Brazil; c Department of Internal Medicine, School of Veterinary Medicine and Animal Science, Universidade de São Paulo, São Paulo, Brazil; d School of Veterinary Medicine, Metropolitan University of Santos, Santos, Brazil; e Laborclin, Pinhais, Brazil; f Natal Garrison Hospital, Brazilian Army, Natal, Brazil; g Department of Clinical Analysis, Health Sciences Center, Federal University of Santa Catarina, Florianópolis, Brazil; h Facultad de Farmacia y Bioquímica, Instituto de Investigaciones en Bacteriologia y Virología Molecular, Universidad de Buenos Aires, Buenos Aires, Argentina; i Department of Pathology, Clinical and Toxicological Analysis, Health Sciences Center, University Hospital of Londrina, Paraná, Brazil; j Fleury Medicine and Health, Microbiology Section, São Paulo, Brazil; Institute of Microbiology, University of Brescia

**Keywords:** carbapenemases, coproduction, avibactam, aztreonam, *K. quasipneumoniae*, *K. variicola*, combined disk test, disk approximation test, immunochromatography

## Abstract

Reports of Gram-negative bacteria harboring multiple carbapenemase genes have increased in South America, leading to an urgent need for appropriate microbiological diagnosis. We evaluated phenotypic methods for detecting Klebsiella pneumoniae carbapenemase 2 (KPC-2) and New Delhi metallo-β-lactamase-1 (NDM-1) coexpression in members of the K. pneumoniae complex (i.e., K. pneumoniae, K. quasipneumoniae, and K. variicola) isolated from human and animal hosts, based on inhibition of ceftazidime-avibactam (CZA) and aztreonam (ATM) by dipicolinic acid (DPA), EDTA, or avibactam (AVI). While the presence of *bla*_KPC-2_ and *bla*_NDM-1_ genes was confirmed by whole-genome sequencing, PCR, and/or GeneXpert, coexpression was successfully detected based on the following: (i) a ≥5-mm increase in the zone diameter of ATM (30 µg) disks plus AVI (4 or 20 µg) and ≥4-mm and ≥10-mm increases in the zone diameters for “CZA 50” (30 µg ceftazidime [CAZ] and 20 µg AVI) and “CZA 14” (10 µg CAZ and 4 µg AVI) disks, respectively, when we added DPA (1 mg/disk) or EDTA (5 mM) in a combined disk test (CDT); (ii) a positive ghost zone (synergism) between ATM (30 µg) and CZA 50 disks and between CZA 50 and DPA (1 mg) disks, using the double-disk synergy test (DDST) at a disk-disk distance of 2.5 cm; (iii) ≥3-fold MIC reductions of ATM and CZA in the presence of AVI (4 µg/mL), DPA (500 µg/mL), or EDTA (320 µg/mL); and (iv) immunochromatography. Although our results demonstrated that inhibition by AVI, DPA, and EDTA may provide simple and inexpensive methods for the presumptive detection of coexpression of KPC-2 and NDM-1 in members of the K. pneumoniae complex, additional studies are necessary to confirm the accuracy of these methodologies by testing other Gram-negative bacterial species and other KPC and NDM variants coexpressed by WHO critical priority pathogens detected worldwide.

**IMPORTANCE** Alerts regarding the emergence and increase of combinations of carbapenemases in *Enterobacterales* in Latin America and the Caribbean have recently been issued by PAHO and WHO, emphasizing the importance of appropriate microbiological diagnosis and the effective and articulated implementation of infection prevention and control programs. In this study, we evaluated methods based on inhibition of ceftazidime (CAZ), ceftazidime-avibactam (CZA), and aztreonam (ATM) by dipicolinic acid (DPA), EDTA, and avibactam (AVI) inhibitors for the identification of KPC-2- and NDM-1-coexpression in members of the K. pneumoniae complex recovered from human and animal hosts. Our results demonstrate that inhibition by AVI, DPA, and EDTA may provide simple and inexpensive methods for the presumptive detection of coexpression of KPC-2 and NDM-1 in members of the K. pneumoniae complex.

## INTRODUCTION

During the COVID-19 pandemic, the incidence of carbapenem-resistant *Enterobacterales* (CRE) has increased in South America and the Caribbean (1, 2). In fact, according to an epidemiological alert of the Pan American Health Organization (PAHO) in October 2021, coexpression of different classes of carbapenemases are expanding in different countries (3). In this regard, coproduction of Klebsiella pneumoniae carbapenemase (KPC) and New Delhi metallo-β-lactamase-1 (NDM-1) seems to be the major threat to public health (1, 3, 4).

Coproduction of KPC-2 and NDM-1 in South America was first detected in Brazil in members of the Enterobacter cloacae complex in 2013 (5). Noteworthy, from 2020 to 2021, coexpression of these enzymes was extended among K. pneumoniae isolates from Argentina, Uruguay, Ecuador, and Paraguay (3), and lately this coexpression has been detected in hospital sewage samples in Brazil (6).

KPC belongs to class A carbapenemases, which share a serine residue at their active site that confers hydrolytic properties (7) that can be inhibited by avibactam (AVI), vaborbactam, and relebactam (8), whereas NDM enzymes belong to class B metallo-beta-lactamases (MβLs), which depend on Zn^2+^ in their catalytic site (9) and can be inhibited by ethylenediaminetetraacetic (EDTA) and dipicolinic acid (DPA) (10). Strikingly, MβLs are unable to hydrolyze aztreonam (ATM) (11). As a result, bacterial species that produce NDM-type MβLs exhibit *in vitro* susceptibility to this antibiotic. However, despite aztreonam not being hydrolyzed by MβLs, frequently such isolates harbor additional cephalosporinases, like AmpC and extended-spectrum β-lactamases (ESBLs).

Phenotypic methods to detect carbapenemases have been based on the use of inhibitors, where an increase in size of the inhibition zone of carbapenem-containing disks is observed by using combined disk (CDT) methods (12, 13), whereas the presence of a ghost zone (synergism) between carbapenem-containing disks and inhibitor-containing disks can be observed by using the double-disk synergy test (DDST) (14). Additionally, production of carbapenemases can be evaluated quantitatively based on the reduction of carbapenem MICs in the presence of specific inhibitors (15). In brief, a modified carbapenem inactivation test (mCIM), colorimetric methods (with Carba NP or Blue Carba), or inhibition tests using synergy with boronic acid or EDTA have also been routinely used, as recommended by CLSI or EUCAST guidelines (16, 17).

In this study, we evaluated methods based on inhibition of ceftazidime (CAZ), ceftazidime-avibactam (CZA), and ATM by DPA, EDTA, and AVI inhibitors for the identification of KPC and NDM coexpression by K. pneumoniae complex members (i.e., K. pneumoniae, K. quasipneumoniae, and K. variicola) recovered from human and animal hosts in South America.

## RESULTS

### Coproduction of NDM-1 and KPC-2 and KPC variants conferring resistance to ceftazidime-avibactam among K. pneumoniae complex members.

Fifteen K. pneumoniae-related species, including K. pneumoniae, K. quasipneumoniae, and K. variicola, displaying resistance to broad-spectrum cephalosporins and ceftazidime-avibactam were identified in human and animal hosts (Table 1). Regarding carbapenem resistance, all isolates were resistant to ertapenem, imipenem, and meropenem, with the exception of K. pneumoniae strains 331 (susceptible to imipenem and meropenem) and MV940851 (susceptible to imipenem).

**TABLE 1:** β-Lactam resistance profiles and carbapenemases and cephalosporinases produced by *Enterobacterales* used in this study

Strain (ST)^*a*^	Origin (yr)	Country	β-Lactam resistance profile^*b*^	Carbapenemase(s)^*c*^	Cephalosporinase(s)^*d*^
K. pneumoniae Kp9417 (ST147)	Human (2021)	Brazil	AMC, CEF, CRO, CAZ, CTX, CFO, CPM, ATM, ETP, IPM, MER, CZA	KPC-2, NDM-1	CTX-M-15
K. pneumoniae Kp9270 (ST147)	Human (2021)	Brazil	AMC, CEF, CRO, CAZ, CTX, CFO, CPM, ATM, ETP, IPM, MER, CZA	KPC-2, NDM-1	None
*K. quasipneumoniae* 795b (ST1308)	Animal (2020)	Brazil	AMC, CEF, CRO, CAZ, CTX, CFO, CPM, ATM, ETP, IPM, MER, CZA	KPC-2, NDM-1	CTX-M-15
*K. quasipneumoniae* 868 (ST1308)	Animal (2020)	Brazil	AMC, CEF, CRO, CAZ, CTX, CFO, CPM, ATM, ETP, IPM, CZA, MER, CZA	KPC-2, NDM-1	CTX-M-15
*K. quasipneumoniae* 883b (ST1308)	Animal (2020)	Brazil	AMC, CEF, CRO, CAZ, CTX, CFO, CPM, ATM, ETP, IPM, CZA, MER, CZA	KPC-2, NDM-1	None
*K. quasipneumoniae* FAI130 (ST1308)	Animal (2020)	Brazil	AMC, CEF, CRO, CAZ, CTX, CFO, CPM, ATM, ETP, IPM, MER, CZA	KPC-2, NDM-1	None
*K. quasipneumoniae* FAI131 (ST1308)	Animal (2020)	Brazil	AMC, CEF, CRO, CAZ, CTX, CFO, CPM, ATM, ETP, IPM, MER, CZA	KPC-2, NDM-1	CTX-M-15
*K. variicola* L221385 (ND)	Human (2019)	Brazil	AMC, CEF, CRO, CAZ, CTX, CFO, CPM, ATM, ETP, IPM, MER, CZA	KPC-2, NDM-1	None
K. pneumoniae 14A (ST437)	Human (2018)	Brazil	AMC, CEF, CRO, CAZ, CTX, CFO, CPM, ATM, ETP, IPM, MER, CZA	KPC-2, NDM-1	None
K. pneumoniae 435AR (ND)	Human (2019)	Argentina	AMC, CEF, CRO, CAZ, CTX, CFO, CPM, ATM, ETP, IPM, MER, CZA	KPC-2, NDM-1	None
K. pneumoniae 338AR (ND)	Human (2019)	Argentina	AMC, CEF, CRO, CAZ, CTX, CFO, CPM, ATM, ETP, IPM, MER, CZA	KPC-2, NDM-1	None
K. pneumoniae MV931658 (ST11)	Human (2019)	Brazil	AMC, CEF, CRO, CAZ, CTX, CFO, CPM, ATM, ETP, IPM, MER	KPC-3	None
K. pneumoniae MV940851 (ST11)	Human (2019)	Brazil	AMC, CEF, CRO, CAZ, CTX, CFO, CPM, ATM, ETP, MER, CZA	KPC-31	None
K. pneumoniae 330 (ST16)	Human (2020)	Brazil	AMC, CEF, CRO, CAZ, CTX, CFO, CPM, ATM, ETP, IPM, MER, CZA	KPC-113	None
K. pneumoniae 331 (ST11)	Human (2020)	Brazil	AMC, CEF, CRO, CAZ, CTX, CFO, CPM, ATM, ETP, CZA	KPC-114	None
K. pneumoniae IBL2.4 (ST11)	Environment (2013)	Brazil	AMC, CEF, CRO, CAZ, CTX, CFO, CPM, ATM, ETP, IPM, MER	KPC-2	None
C. freundii PG4 (ST214)	Animal (2020)	Brazil	AMC, CEF, CRO, CAZ, CTX, CFO, CPM, ETP, IPM, MER, CZA	NDM-1	CMY-48
K. pneumoniae Kp183 (ST1639)	Human (2017)	Brazil	AMC, CEF, CRO, CAZ, CTX, CFO, CPM, ATM, ETP, IPM, MER, CZA	NDM-1	CTX-M-15
E. coli 2ECMBL (ST155)	Human (2017)	Peru	AMC, CEF, CRO, CAZ, CTX, CFO, CPM, ATM, ETP, IPM, MER, CZA	NDM-1	PER-2
K. pneumoniae PRETA (ST307)	Animal (2018)	Brazil	AMC, CEF, CRO, CAZ, CTX, CFO, CPM, ATM	None	CTX-M-15, SHV-28
E. coli Em1cro (ST457)	Animal (2016)	Brazil	AMC, CEF, CFO, ATM	None	CMY-2

a ST, sequence type predicted by MLST 2.0 (https://cge.food.dtu.dk/services/MLST/); ND, not determined.

b Resistance profile determined by disk diffusion, Vitek 2, or broth microdilution methods. AMC, amoxicillin-clavulanic acid; CEF, cephalothin; CRO, ceftriaxone; CAZ, ceftazidime; CTX, cefotaxime; CFO, cefoxitin; CPM, cefepime; ATM, aztreonam; ETP, ertapenem; IPM, imipenem; MER, meropenem; CZA, ceftazidime-avibactam.

c Detected by PCR, GeneXpert, immunochromatography, and/or WGS.

d Detected by PCR and/or WGS.

**FIG 1:**
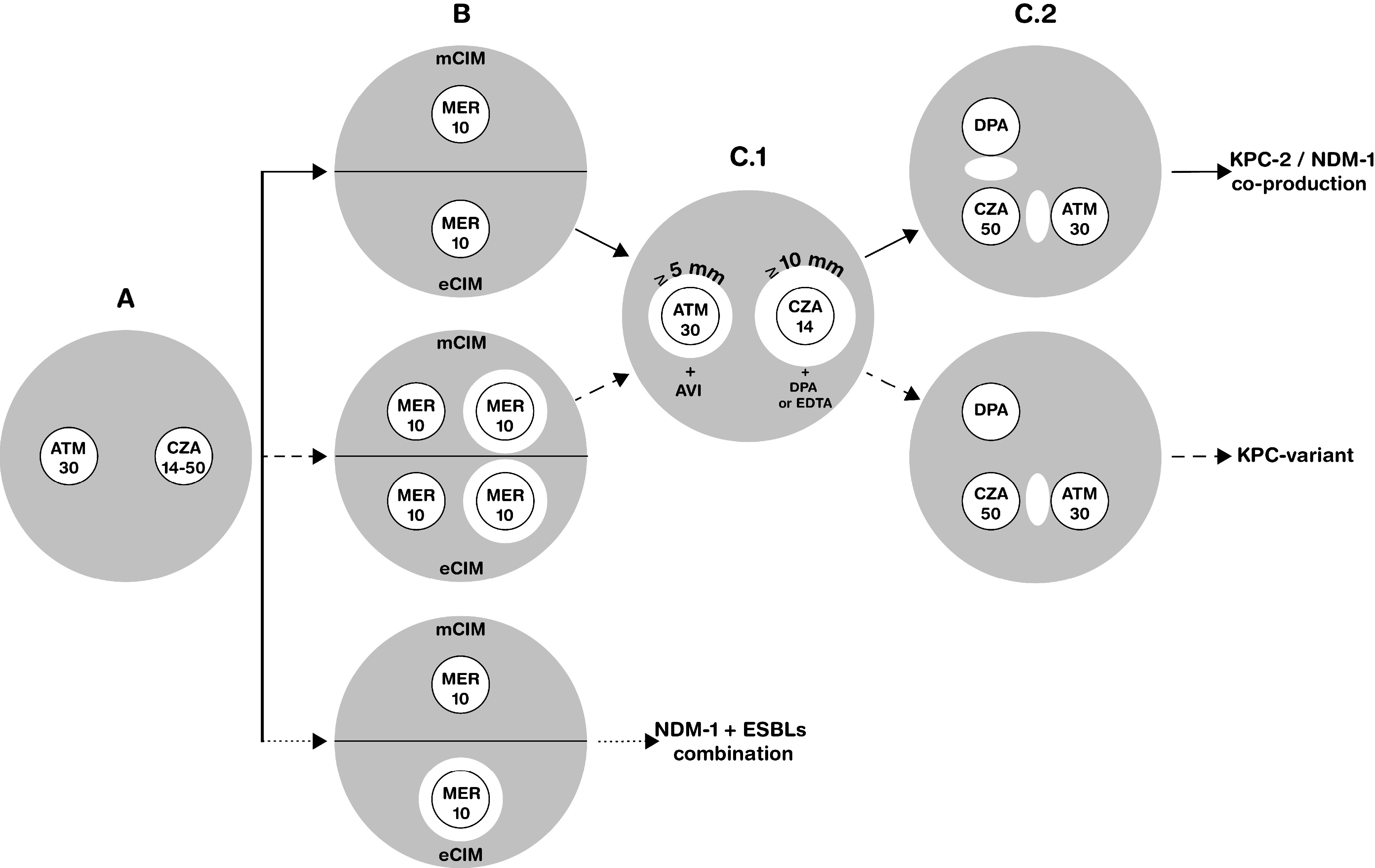
Workflow proposed for identification of KPC-2 and NDM-1 coexpression in members of the K. pneumoniae complex. (A) Isolates displaying resistance to ATM (30 µg/disk) and CZA 14 (ceftazidime at 10 µg/disk, avibactam at 4 µg/disk) or 50 (ceftazidime 30 µg/disk, avibactam 20 µg/disk) were submitted for mCIM and eCIM tests. (B) Isolates coexpressing KPC-2 and NDM-1 exhibited positive mCIM and negative eCIM results (solid arrow). Variable mCIM and eCIM results are indicative of the presence of KPC variants conferring resistance to CZA (dashed arrow). Positive mCIM and eCIM results indicated the presence of NDM-1 and ESBL coexpression (dotted arrow). (C.1) In the combined disk test (CDT), a ≥5 mm increase in the zone diameter of ATM (30 µg) disks plus AVI (4 or 20 µg), and a ≥4 or ≥10 mm increase in the zone diameter of CZA 50 and CZA 14 disks, respectively, when added DPA (1 mg/disk) or EDTA (5 mM) was added, was indicative of KPC-2 and NDM-1 coproduction or a KPC variant conferring resistance to CZA. (C.2) By using the double-disk synergy test (DDST), a positive ghost zone (synergism) between ATM (30 µg) and CZA (50 µg) disks and between CZA (50 µg) and DPA (1 mg) disks, at a disk-disk distance of 2.5 cm, was indicative of KPC-2 and NDM-1 coproduction (solid arrow), whereas a positive ghost zone between ATM (30 µg) and CZA (50 µg) disks alone was indicative of KPC variant conferring resistance to CZA (dashed arrow).

Initially, metallo-β-lactamase and serine carbapenemase production was screened by using mCIM and EDTA-modified carbapenem inactivation (eCIM) (Fig. 1). In this regard, while 13 Klebsiella spp. showed mCIM^+^ eCIM^−^ results, 2 K. pneumoniae strains displayed an indeterminate result (i.e., mCIM^−^ eCIM^−^). It is important to emphasize that eCIM is not an accurate method to detect suspected coproduction of class A and class B carbapenemases, as it only detects MβLs if both the mCIM and eCIM are positive, whereas mCIM^+^ eCIM^+^ results may be caused by NDM or MβLs plus AmpC.

Strikingly, immunochromatography revealed coproduction of NDM- and KPC-type carbapenemases in 11 members of the K. pneumoniae complex, whereas 2 of 4 CZA-resistant K. pneumoniae strains displayed positive bands for KPC production alone (see Table S1 in the supplemental material). Coproduction of NDM and KPC was confirmed by PCR and/or GeneXpert, and further genomic analysis predicted *bla*_KPC-2_ and *bla*_NDM-1_ genes. On the other hand, genomic analysis of four CZA-resistant K. pneumoniae strains confirmed the presence of *bla*_KPC-3_, *bla*_KPC-31_, *bla*_KPC-113_, and *bla*_KPC-114_ variants. Expression of KPC-31 and KPC-114 was not detected by immunochromatography.

### Detection of KPC-2 and NDM-1 coexpression by the combined disk test.

For the CDT, with different EDTA and DPA concentrations tested, 5 mM EDTA/disk and 1,000 µg DPA/disk were chosen for inhibition activity of MβL, since these concentrations showed no inhibitory activity across the bacterial growth of all screened isolates when sterile blank disks impregnated with 10 μL of 0.1 M EDTA and 10 mg/mL DPA were tested. On the other hand, in order to detect expression of NDM enzymes, ceftazidime-avibactam at 14 μg/disk (10 μg CAZ and 4 μg AVI; “CZA 14”) and 50 μg/disk (30 μg CAZ and 20 μg AVI; “CZA 50”) were used, in accordance with guidelines for disk-diffusion antimicrobial susceptibility tests of EUCAST and CLSI, respectively.

**FIG 2:**
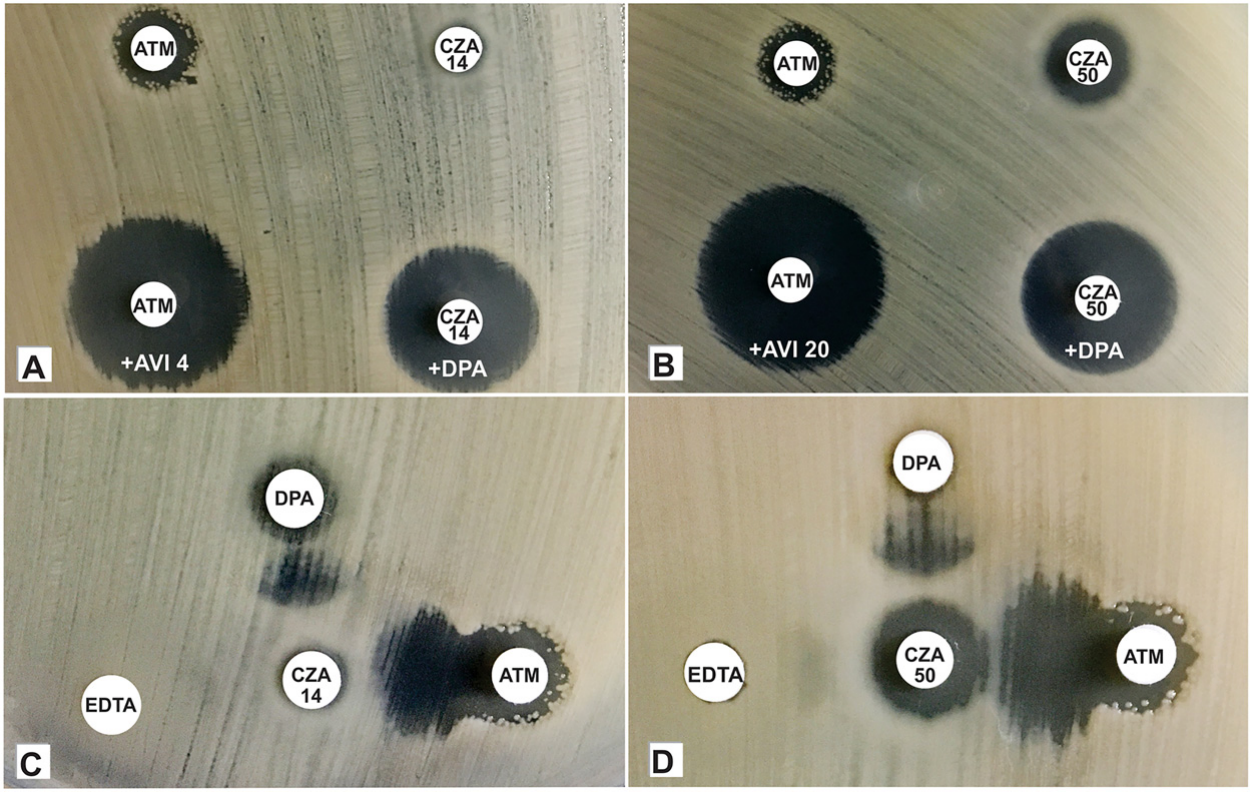
Positive results of a combined disk test (CDT) and double-disk synergy test (DDST) for K. pneumoniae strain FAI131 coproducing KPC-2 and NDM-1 carbapenemases. (A) A ≥5-mm increase in the zone diameter of an ATM (30 µg) disk plus AVI (4 µg) and of a ≥10-mm increase in the zone diameter of a CZA 14 disk plus DPA (1 mg/disk) was observed in the CDT. (B) A ≥5-mm increase in the zone diameter of ATM (30 µg) disk plus AVI (20 µg) and of a ≥4-mm increase in the zone diameter of a CZA 50 disk plus DPA (1 mg/disk) was observed in the CDT. (C) A positive ghost zone (synergism) between ATM (30 µg) and CZA (14 µg) disks, between CZA (14 µg) and DPA (1 mg) disks, and a negative ghost zone between CZA (14 µg) and EDTA (5 mM) disks, at a disk-disk distance of 2.5 cm, was observed in the DDST. (D) A positive ghost zone (synergism) between ATM (30 µg) and CZA (50 µg) disks, between CZA (50 µg) and DPA (1 mg) disks, and a negative ghost zone between CZA (50 µg) and EDTA (5 mM) disks, at a disk-disk distance of 2.5 cm, was observed in the DDST.

For KPC-2-positive NDM-1-positive K. pneumoniae complex isolates, an increase of ≥10 mm in the size of inhibition zones were observed around CZA 14 disks containing 10 µL of 0.1 M EDTA or 10 µL of 10-mg/mL DPA, in comparison to the inhibition zones of CZA disks without EDTA or DPA. Otherwise, for the same KPC-2^+^ NDM-1^+^ isolates, increases of ≥4 mm in the size of inhibition zones were observed around CZA 50 disks containing 10 µL of 0.1 M EDTA or 10 µL of 10-mg/mL DPA, in comparison to the inhibition zones of CZA disks without EDTA or DPA. Additionally, for KPC-2 and NDM-1 coproducers, increases of ≥5 mm in the size of inhibition zones were observed around the ATM 30-μg AVI 4-μg disks, in comparison to the inhibition zones of ATM disks without AVI (Fig. 2A and B). Exceptionally, two CZA-resistant K. pneumoniae isolates (strains 330 and 331), which were NDM-1 negative and carried *bla*_KPC-113_ or *bla*_KPC-114_ gene variants, exhibited an increase of ≥10 mm in the inhibition zones around CZA-DPA and displayed no increase in the inhibition zones around CZA-EDTA disks. On the other hand, the *bla*_KPC-31_-positive K. pneumoniae strain MUV940851 displayed an increase of ≥10 mm in the inhibition zones around CZA-DPA and CZA-EDTA disks, while no increase around the ATM-AVI disk was detected, supporting the prediction that coproduction of KPC-2 and NDM-1 must be based on a positive synergistic effect shown using both CZA-EDTA and ATM-AVI disk combinations and not a single combination in the CDT test.

While for ATM-AVI disks a negative synergistic activity was expected against the KPC-31 producer, due to its resistance to CZA, positive synergistic activities against KPC-113- and KPC-114-producing K. pneumoniae strains suggested that combinations of monobactams and AVI produced inhibitory effects on some KPC variants, in a similar way as for NDM-type carbapenemases. In this respect, since AVI is able to covalently bind to some bacterial penicillin-binding proteins (PBPs), synergistic activity of ATM-AVI against KPC-113 and KPC-114 may be related with different activities on multiple PBP targets (18). It is important also to emphasize that despite there being an “*in vitro* synergy” between ATM-CZA disks in KPC variants resistant to CZA, this doesn't mean that the combination is clinically active. Finally, synergistic activity of CZA-DPA against KPC-113- and KPC-114-producing K. pneumoniae strains deserves additional investigation.

### Detection of KPC-2 and NDM-1 coexpression by the double-disk synergy test.

For all KPC-2^+^ NDM-1^+^ isolates (*n =* 11), a positive ghost zone (synergism) was observed between CZA 50 and ATM disks and between CZA 50 and DPA disks, with disks positioned at a disk-disk distance of 2.5 cm (Table 2; Fig. 2D). On the other hand, while all KPC-2^+^ NDM-1^+^ isolates exhibited a ghost zone between CZA 14 and DPA disks (Fig. 2C), only 9 KPC-2^+^ NDM-1^+^ isolates exhibited a positive ghost zone between CZA 14 and ATM disks, with disks positioned at a disk-disk distance of 2.5 cm (Table 2). For all KPC-2^+^ NDM-1^+^ isolates, a negative ghost zone between EDTA and CZA 14 disks was observed, whereas 4 KPC-2 and NDM-1 coproducing isolates showed a positive ghost zone between EDTA and CZA 50 disks, with disks positioned at a disk-disk distance of 2.5 cm (Table 2). Otherwise, while 5 KPC-2 and NDM-1 coproducing isolates showed a positive ghost zone between CZA 14 and EDTA disks, 8 KPC-2 and NDM-1 coproducing isolates showed a positive ghost zone between CZA 50 and EDTA disks at a disk-disk distance of 1.5 cm (Table 2).

**TABLE 2:** Detection of KPC-2 and NDM-1 coproduction in K. pneumoniae complex-related species

Strain	Resistance^*a*^						CDT^*b*^								DDST^*c*^										mCIM^*d*^	eCIM^*d*^
	EUCAST			CLSI			ATM + AVI 4	ATM + AVI 20		CZA 14 + DPA	CZA 14 + EDTA	CZA 50 + DPA	CZA 50 + EDTA		CZA 14					CZA 50						
	ATM	CZA 14		ATM	CZA 50										ATM	DPA	EDTA	EDTA*		ATM	DPA	EDTA	EDTA*			
K. pneumoniae Kp9417																										
K. pneumoniae Kp9270																										
*K. quasipneumoniae* 795b																										
*K. quasipneumoniae* 868																										
*K. quasipneumoniae* 883b																										
*K. quasipneumoniae* FAI130																										
*K. quasipneumoniae* FAI131																										
*K. variicola* L221385																										
K. pneumoniae 14A																										
K. pneumoniae 435AR																										
K. pneumoniae 338AR																										
K. pneumoniae MV931658																										
K. pneumoniae MV940851																										
K. pneumoniae 330																										
K. pneumoniae 331																										
K. pneumoniae IBL2.4																										
C. freundii PG4																										
K. pneumoniae Kp183																										
E. coli 2ECMBL																										
K. pneumoniae PRETA																										
E. coli Em1cro																										

a Gray squares indicate resistance. White squares indicate susceptibility. ATM, aztreonam; CZA-14, ceftazidime at 10 µg/disk and avibactam at 4 µg/disk; CZA 50, ceftazidime at 30 µg/disk and avibactam at 20 µg/disk.

b Gray squares indicate a positive result for the test; white squares indicate a negative result for the test. A ≥4-mm or ≥10-mm increase in the zone diameter of CZA 50 and CZA 14 disks, respectively, in the presence of DPA (1 mg/disk) or EDTA (5 mM) was interpreted as a positive CDT result. ATM, aztreonam; AVI 4, avibactam 4 µg/disk; AVI 20, avibactam 20 µg/disk; CZA 14, ceftazidime at 10 µg/disk and avibactam at 4 µg/disk; CZA 50, ceftazidime 30 µg/disk and avibactam 20 µg/disk; DPA, dipicolinic acid.

c DDST, double-disk synergy test. Gray squares indicate a positive result for the test; white squares indicate a negative result for the test. A positive ghost zone (synergism) between ATM (30 µg) and CZA (14 or 50 µg) disks, CZA and DPA (1 mg) disks, and CZA and EDTA (5 mM) disks was interpreted as a positive DDST result. CZA (14 or 50) disks were placed 2.5 cm center to center from DPA (1,000 µg), EDTA (5 mM), and ATM (30) disks. *, EDTA (5 mM) and CZA (14 or 50) disks tested at 1.5 cm center to center.

d Modified carbapenem inactivation (mCIM) and EDTA-modified carbapenem inactivation (eCIM) tests. Gray squares indicate a positive result for the test. White squares indicate a negative result for the test.

Although NDM-1^+^ CTX-M-15^+^ K. pneumoniae KP183 and NDM-1^+^ PER-2^+^ Escherichia coli 2ECMBL control strains displayed a positive ghost zone between CZA and ATM and between CZA and DPA disks, it is very important to highlight that this positive DDST result was related to ESBL production, since these enzymes hydrolyze ATM, which is inhibited by AVI. In fact, the positive mCIM and eCIM results displayed by these strains confirmed production of MβL alone, as it was not necessary to perform CDT and DDST for NDM-1 KPC-2 coproduction. Therefore, for both CDT and DDST interpretation, we highly recommended the following conditions: (i) ATM and CZA resistance is observed; (ii) KPC-2 and NDM-1 coproducers are mCIM^+^ and eCIM^−^, and (iii) KPC variants conferring resistance to CZA could be susceptible to meropenem, displaying an indeterminate mCIM and eCIM result, as interpreted by CLSI guidelines. All CDT and DDST results are summarized in Table 2; see also Table S2.

### Reduction of aztreonam and ceftazidime-avibactam MICs in the presence of AVI, EDTA, or DPA as an indicator of KPC-NDM coproduction.

For MIC reduction assays, the final concentrations of EDTA, DPA, and AVI were fixed at 320, 500, and 4 µg/mL, respectively, since these concentrations produced no antibacterial activity against any screened isolates, allowing us to observe a ≥3-fold decrease in ATM and CZA MICs among NDM-1 and KPC-2 coproducers in the presence of inhibitors. In Table 3 and Table S3, results of reproducible replicates, performed three times on three distinct occasions, are shown.

**TABLE 3:** MIC reductions induced by AVI, DPA, and EDTA for detection of NDM-1 and KPC-2 coproduction in K. pneumoniae complex-related species

Strain	MIC (µg/mL)^*a*^					
	ATM	ATM + AVI	CAZ	CZA	CZA + DPA	CZA + EDTA
K. pneumoniae Kp9417	>256	0.25	>256	>256	0.5	0.25
K. pneumoniae Kp9270	>256	0.25	>256	>256	0.25	0.25
*K. quasipneumoniae* 795b	>256	0.25	>256	>256	0.25	0.125
*K. quasipneumoniae* 868	>256	0.25	>256	>256	0.25	0.25
*K. quasipneumoniae* 883b	>256	0.25	>256	256	0.125	1
*K. quasipneumoniae* FAI130	>256	0.25	>256	>256	0.25	0.25
*K. quasipneumoniae* FAI131	>256	0.25	>256	>256	0.25	0.25
*K. variicola* L221385	>256	0.25	>256	>256	0.25	0.25
K. pneumoniae 14A	8	0.25	>256	>256	0.5	0.25
K. pneumoniae 435AR	>256	2	>256	>256	0.5	0.5
K. pneumoniae 338AR	>256	0.25	>256	>256	0.25	0.125
K. pneumoniae MV931658	>256	0.25	>256	8	0.5	1
K. pneumoniae MV940851	16	0.25	>256	>256	64	16
K. pneumoniae 330	>256	0.25	>256	64	0,5	4
K. pneumoniae 331	>256	0.25	256	64	8	0.125
K. pneumoniae IBL2.4	>256	0.25	128	2	0.25	0.25
C. freundii PG4	4	0.25	>256	>256	<0.5	0.25
K. pneumoniae Kp183	128	0.25	>256	>256	<0.5	0.25
E. coli 2ECMBL	128	1	>256	>256	<0.5	0.25
K. pneumoniae PRETA	64	0.25	32	0.25	0.25	0.25
E. coli Em1cro	8	0.25	32	0.25	0.25	0.25

a MICs were determined by broth microdilution method according to CLSL and EUCAST guidelines (18, 19). The MIC reduction of ATM (aztreonam), CAZ (ceftazidime), and CZA (ceftazidime-avibactam) was evaluated in the presence of avibactam (AVI; 4 µg/mL), dipicolinic acid (DPA; 500 µg/mL), and EDTA (320 µg/mL). All assays were performed in triplicate on distinct dates.

## DISCUSSION

The emergence of carbapenem-resistant clinical isolates has become a serious clinical challenge due to the limited treatment options, and the coproduction of multiple carbapenemases by isolates aggravates this issue. There are only limited effective antibiotics against such strains. Combinations of CZA with meropenem and colistin seem to show potential synergism against these isolates. On the other hand, combinations of ATM plus meropenem-vaborbactam or plus CZA have demonstrated synergy against MβL and ATM-resistant NDM-producing *Enterobacterales*. Thus, the combination of aztreonam plus avibactam appears to be a promising option against *Enterobacterales* isolates coproducing class A and class B β-lactamases while awaiting development of new antimicrobials (19–24).

Epidemiological alerts have been released by PAHO and WHO in view of the emergence and increase of clinically relevant carbapenem-resistant bacteria coproducing KPC and NDM β-lactamases in Latin America and the Caribbean, which has been related to the increased use of broad-spectrum antibiotics in patients with COVID-19. These concerns emphasize the importance of appropriate microbiological diagnosis and the effective and articulated implementation of infection prevention and control programs (3, 25).

In this study, we identified 15 carbapenem- and CZA-resistant isolates belonging to the K. pneumoniae complex, of which 11 coproduced NDM-1 and KPC-2 carbapenemases. Since conventional phenotypic methods failed to detect serine carbapenemase and MβL coproduction, we tested modifications of the DDST and CDT methods based on use of avibactam, EDTA, and DPA as inhibitors, with aztreonam and ceftazidime-avibactam as enzymatic substrates. These modifications were carried out considering that MβLs (including NDM-1) are susceptible to aztreonam and are inhibited by EDTA or DPA (11, 26), whereas KPC-2 serine carbapenemases are susceptible to CZA and are inhibited by AVI (27). Indeed, we observed that Klebsiella isolates coproducing KPC-2 and NDM-1 displayed a positive CDT, with ≥4-mm inhibition zones around CZA 50 with DPA or CZA 50 with EDTA disks and ≥5-mm inhibition zones around ATM-AVI disks containing 4 µg/mL AVI. For CZA 14 with DPA or CZA 14 with EDTA disks, a ≥10-mm inhibition zone was defined as indicative of NDM-1 production. In Fig. 1, a workflow for detection of NDM-1 and KPC-2 coproduction in *Enterobacterales* is proposed.

Since all isolates coproducing NDM-1 and KPC-2 displayed a positive ghost zone in the DDST, by using CZA 50-ATM and CZA 50-DPA disk combinations, at a 2.5-cm disk-disk distance, it was evident that use of DPA was more efficient than EDTA, even when a 1.5-cm disk-disk distance was used for CZA 50-EDTA disk combinations, as previously suggested (28). On the other hand, for the DDST, use of a CZA 14 disk is not recommended. All these results were confirmed based on ≥3-fold reductions of aztreonam and ceftazidime-avibactam MICs in the presence of the inhibitors AVI, EDTA, or DPA.

Although CZA-resistant isolates producing KPC variants displayed a positive CDT with CZA-DPA or CZA-EDTA disks, similar to MβL producers, it is important to consider that these isolates presented indeterminate mCIM and eCIM results, which could be associated with low resistance levels for meropenem (29–31). In fact, it has been reported that some CZA-resistant Klebsiella spp. producing KPC variants display susceptibility or low MICs to imipenem and/or meropenem (29–31). On the other hand, these CZA-resistant KPC variants can be presumptively detected by DDST, where a positive ghost zone was observed between ATM and CZA disks and no ghost zone observed between CZA and DPA disks. In brief, it is important to test both CZA and ATM to detect KPC variants or carbapenemase-coproducing organisms, even if in some countries those drugs are not used for clinical treatment. Likewise, strains that are mCIM^+^ eCIM^+^ and resistant to aztreonam should go through testing to rule out additional enzymes.

Isolates coproducing NDM-1 and ESBLs could show positive CDT and DDST results for ATM-AVI and CZA-ATM combinations. However, it is important to highlight that positive mCIM and eCIM tests must be observed for NDM-1 and ESBL coproducers, whereas a positive mCIM and a negative eCIM must be observed for NDM-1^+^ KPC-2^+^ strains.

Although immunochromatography methods can rapidly detect coproduction of KPC and NDM carbapenemases, they can fail to identify variants and other combinations of carbapenemases, such as Australian imipenemase, Guiana extended-spectrum β-lactamase, German imipenemase, imipenem-hydrolyzing β-lactamase, Seoul imipenemase, Serratia marcescens extended-spectrum β-lactamase, and/or São Paulo metallo-β-lactamase (32). In addition, immunochromatography methods are more expensive than other methods (33). Otherwise, methods based on disk combinations, disk elution, and disk prediffusion are valuable and useful in low-resource settings that routinely use disk diffusion for susceptibility testing due to affordability (12, 34, 35). Specifically, CDT and DDST methods have strong potential to identify KPC variants and other combinations of carbapenemases that are undetectable by immunochromatography methods (32, 36, 37). Moreover, the inclusion of a CZA-ATM combination in CDT and DDST methods also has clinical significance because this combination has shown effectiveness against pathogens coproducing carbapenemases (38–41). However, disadvantages of CDT and DDST can include the long turnaround time for results. Since the detection of rare carbapenemases is still problematic with most of the commercially available tests, the combination of methods will enable most laboratories to detect these rare variants and, along with performing accurate antimicrobial susceptibility testing, this could help to optimize patient treatment and limit the further spread of carbapenemase producers (36).

In this study, immunochromatography did not detect KPC-31-positive or KPC-114-positive isolates exhibiting resistance to CZA, which could be a limitation of this method. Therefore, for CZA-resistant isolates, additional testing is recommended. On the other hand, a limitation of this study is the reduced numbers of isolates coproducing NDM and KPC tested and the lack of isolates showing coproduction mediated by other MβL and KPC variants. However, this limitation is due to the recent observation of coproduction phenomena in Latin America. Nevertheless, our results demonstrate that inhibition by AVI, DPA, and EDTA may provide simple and inexpensive methods for the presumptive detection of coexpression of KPC and NDM in members of the K. pneumoniae complex in human and veterinary diagnostic laboratories. Therefore, additional studies are necessary to confirm the accuracy of these methodologies by testing other Gram-negative bacterial species or other KPC- and NDM-coexpressing variants. Moreover, further studies should be performed using different brands of disks and with Mueller-Hinton agar. Finally, since class A and class B carbapenemases may travel together as well in mobile genetic elements (42–45), clinical laboratories should test such strains by using those methodologies to demonstrate accuracy, whereas measures should be taken to closely monitor and control the spread of critical priority WHO pathogens coproducing carbapenemases worldwide.

### Conclusion.

In recent years, several studies have reported the emergence of pathogens coproducing multiple carbapenemases. In this regard, while coproduction of OXA-48 and NDM-1 has been previously reported (46, 47), coproduction of KPC-2 and NDM-1 among K. pneumoniae isolates increased during the COVID-19 pandemic as a major challenge for clinical laboratories (3, 4, 19, 43–45). CZA has demonstrated both excellent *in vitro* and *in vivo* activities against class A carbapenemase producers. However, there is increasing evidence of *in vivo* selection of CZA-resistant strains that have developed mutations in KPC, AmpC, CTX-M, OXA-48, VEB, and/or PER β-lactamases (48, 49). Therefore, guidelines regarding methods to screen coproduction of carbapenemases and variants of enzymes conferring resistance to CZA require an urgent update, especially after the SARS-CoV-2 pandemic. In regions and hospitals with high circulation of KPC mutants, genomic investigation is highly recommended. If such tools are not available, resistance profiles to CZA and ATM using traditional antimicrobial susceptibility testing and screening using inhibition by AVI and DPA could be a viable alternative.

## MATERIALS AND METHODS

### Bacterial isolates, identification, and susceptibility profiles.

From 2018 to 2021, 15 carbapenem-resistant and/or ceftazidime-avibactam-resistant isolates belonging to the K. pneumoniae complex were recovered from human and animal hosts (Table 1). Initially, identification and susceptibility profiles were obtained by use of matrix-assisted laser desorption ionization–time of flight (Bruker), and Vitek-2 (bioMérieux) instruments and disk diffusion methods, respectively. Specifically, ceftazidime-avibactam (CAZ-AVI) disks (Liofilchem) containing CZA 14 and CZA 50 were tested and interpreted according to EUCAST and CLSI breakpoints, respectively (50, 51). E. coli ATCC 25922 and K. pneumoniae ATCC 700603 were used as control strains.

### Carbapenemase detection.

The presence of carbapenemase-encoding genes was evaluated by whole-genome sequencing (WGS) with an Illumina NextSeq platform and/or by GeneXpert (52), or by PCR methods using specific primers (53). Expression of KPC and/or NDM carbapenemases was evaluated by using the modified carbapenem inactivation (mCIM) and EDTA-modified carbapenem inactivation (eCIM) assays (54) and by the NG-Test Carba 5 (NG Biotech, Guipry, France) immunochromatographic method.

### CDT and DDST for detection of KPC and NDM coproduction.

Both the CDT and DDST were adapted from methods previously described for the detection of carbapenemases (55). ATM 30, CZA 14, or CZA 50 disks were used as substrates for carbapenemase activity, whereas EDTA and DPA were used as inhibitors of MβL activity and AVI was used as an inhibitor of KPC activity. In brief, while 10 μL of 100 mM EDTA or 10 μL of 10 mg/mL DPA was added to CZA (14 and 50) disks (56, 57), 10 μL of 400 or 2,000 µg/mL AVI was added to ATM disks. In this way, for each screened isolate, ATM disks without and with AVI (4 or 20 µg/disk) and CZA disks without and with EDTA or DPA were placed onto Mueller-Hinton agar plates (Becton, Dickinson, Le Pont de Claix, France) previously inoculated with a 0.5 McFarland standard bacterial suspension (Fig. 1). Inhibition zone diameters around the antibiotic disks (with and without EDTA, DPA, or AVI) were measured and compared after 18 to 24 h of incubation at 37°C. Blank disks containing 5 mM EDTA, 1,000 µg DPA, or 4 or 20 µg AVI were used as controls. For the DDST, CZA (14 or 50) disks were placed 2.5 cm apart (center to center) from DPA (1,000 µg), EDTA (5 mM), and ATM (30 µg) disks onto Mueller-Hinton agar plates previously inoculated (Fig. 1). Additionally, EDTA and CZA disks were placed 1.5 cm apart (center to center), as previously suggested (28). Results were analyzed 18 to 24 h after incubation at 37°C. Isolates previously characterized by WGS as KPC-2 (K. pneumoniae IBL2.4), NDM-1 and CMY-48 (Citrobacter freundii PG4), NDM-1 and CTX-M-15 (K. pneumoniae Kp183), NDM-1 and PER-2 (Escherichia coli 2ECMBL), CTX-M-15 and SHV-28 (K. pneumoniae PRETA), and CMY-2 (E. coli Em1cro) producers were used as controls (Table 1). All assays were performed in triplicate on distinct dates.

### MIC reductions in the presence of EDTA, DPA, or AVI.

For MIC determinations, ATM, CAZ, EDTA, and DPA were purchased from Sigma-Aldrich, and avibactam was purchased from Selleckchem. All MICs were determined by the broth microdilution methodology outlined in ISO 20776 (50, 51). In brief, bacterial inoculum was adjusted to a 0.5 McFarland turbidity standard and diluted to a ratio of 1:10 in Mueller-Hinton broth (Becton, Dickinson, France). All isolates were tested in serial dilutions of ATM and CAZ, ranging from 0.06 to 256 μg/mL. For MIC reduction assays, the final concentrations of EDTA and DPA were fixed at 320 and 900 μg/mL, respectively, since these concentrations showed no antibacterial activity against any of the screened isolates. Avibactam was tested at a final concentration of 4 µg/mL. E. coli ATCC 25922 and K. pneumoniae ATCC 700603 were used as susceptible controls (Table 3). All assays were performed in triplicate on distinct dates. MIC interpretation was performed according to CLSI and EUCAST breakpoints (50, 51).
